# Undeveloped till soils in scree areas are an overlooked important phosphorus source for waters in alpine catchments

**DOI:** 10.1038/s41598-023-42013-4

**Published:** 2023-09-07

**Authors:** Jiří Kaňa, Eva Kaštovská, Michal Choma, Petr Čapek, Karolina Tahovská, Jiří Kopáček

**Affiliations:** 1 Institute of Hydrobiology, Biology Centre CAS, Na Sádkách 7, 37005 České Budějovice, Czech Republic; 2 Department of Ecosystem Biology, Faculty of Science, University of South Bohemia in České Budějovice, Branišovská 1645/31a, 370 05 České Budějovice, Czech Republic

**Keywords:** Biogeochemistry, Carbon cycle, Element cycles, Environmental chemistry

## Abstract

Scree deposits in alpine catchments contain undeveloped till soils that are “hidden” between and under stones. These scree areas have no vegetation except for sparse lichen patches on stone surfaces, but the soils exhibit biological activity and active cycling of nitrogen (N), phosphorus (P), and organic carbon (C). We compared the chemical and biochemical properties of till soils in the scree areas (scree soils) with developed soils in alpine meadows (meadow soils) of 14 catchments in the alpine zone of the Tatra Mountains. The data showed that scree soils served as an important source of mobile P forms for waters in high elevation catchments. We then conducted a detailed soil survey focused on four selected alpine catchments with scree cover proportions > 30%. This study confirmed that scree soils have significantly higher concentrations of mobile P forms compared to meadow soils, and a high specific microbial activity directed towards the extraction of P with rapid turnover in the microbial biomass. The combination of these properties and the amounts of scree soils in high-elevation areas highlight their importance in overall biogeochemical P cycling in alpine catchments, and the terrestrial P export to receiving waters.

## Introduction

Alpine catchments in the Tatra Mountains (Slovak-Polish border; central Europe) represent relatively remote ecosystems without direct human impacts, except for acidification and N-saturation caused by the long-distance transport of sulphur and nitrogen (N) compounds that peaked in the 1980s and has been dramatically decreasing since the early 1990s^1^. As a response to the decreasing acidic deposition, lake water chemistry and biota (regularly studied since 1984) have been successfully recovering^2–4^. At the same time, increases in dissolved organic carbon (DOC), total organic N (TON), and total phosphorus (P) concentrations have been recently observed in the Tatra Mountain lakes^5^. Similarly, P concentrations have been rising in some other European^6^ and North American^7–9^ mountain waters. P usually limits the primary production of mountain lakes, and elevated P inputs thus increase their algal concentrations and change the composition of zooplankton and benthos, altering anticipated trajectories during recovery from their original pre-acidification states^4^. The changes in P inputs to the Tatra Mountain lakes are probably associated with the recovery of till soils in scree areas (hereafter scree soils) from acidification (increasing P co-export with DOC)^10^ and climatic changes, affecting both the physico-chemical weathering of rocks and soil microbial processes in the catchments^11–13^.

Differences in the recovery rates and trajectories of chemical and biological parameters among the Tatra Mountain lakes reflect the importance of the terrain cover of their catchments such as areal proportions of meadows and scree deposits^5^. The chemical composition of lake water reflects (and integrates) biogeochemical processes in the catchment, which serves as a “transfer medium” of elements (originating from atmospheric deposition and weathering) during their transport from terrestrial to aquatic ecosystems. Alpine catchments widely differ in their proportions of meadows, scree deposits, and bare rocks. Recently, lakes within catchments with abundant scree areas have exhibited faster increases in pH and in concentrations of bicarbonate, calcium (Ca), P, DOC, and TON than lakes in catchments with larger proportions of alpine meadows^5,12^. This indicates that scree areas may be an important source of Ca, bicarbonate and P for lakes, while patchy areas of meadows may more effectively immobilize nutrients entering the catchments with atmospheric deposition or originating from weathering. In accord with this, previous studies on alpine Tatra Mountain soils have shown that scree soils have higher concentrations of extractable P forms^14^ and a higher ability to liberate phosphate during their pH increase than soils in alpine meadows (hereafter meadow soils)^10^.

Direct atmospheric deposition on surfaces is an important source of P for lakes, especially for those with a small ratio of catchment to lake area. The P concentrations in atmospheric deposition are an order of magnitude higher than in most of the Tatra Mountain alpine lakes^15^. This atmospherically deposited P is more effectively immobilized in soils than in bare rock areas. Consequently, the proportion of rocks, meadows and scree deposits in catchments is also important for the overall catchment ability to immobilize atmospherically deposited P.

Besides physico-chemical processes, phosphorus in soils is also immobilized by plants and transformed into organic forms (e.g. Ref.^16^) or into the microbial biomass (e.g. Refs.^17,18^); both of these processes are expected to be higher in vegetated parts of the catchment.

The aim of this study was to characterize the chemical and microbial characteristics of scree soils and compare them with alpine meadow soils, with an emphasis on their potential role in P cycling. Besides their lower ability of scree soils to adsorb atmospherically deposited P and higher ability of P desorption during pH increases, we hypothesized that higher P leaching from scree than meadow soils resulted from their lower potential to immobilize P in soil organic matter and microbial biomass.

## Materials and methods

**Figure 1:**
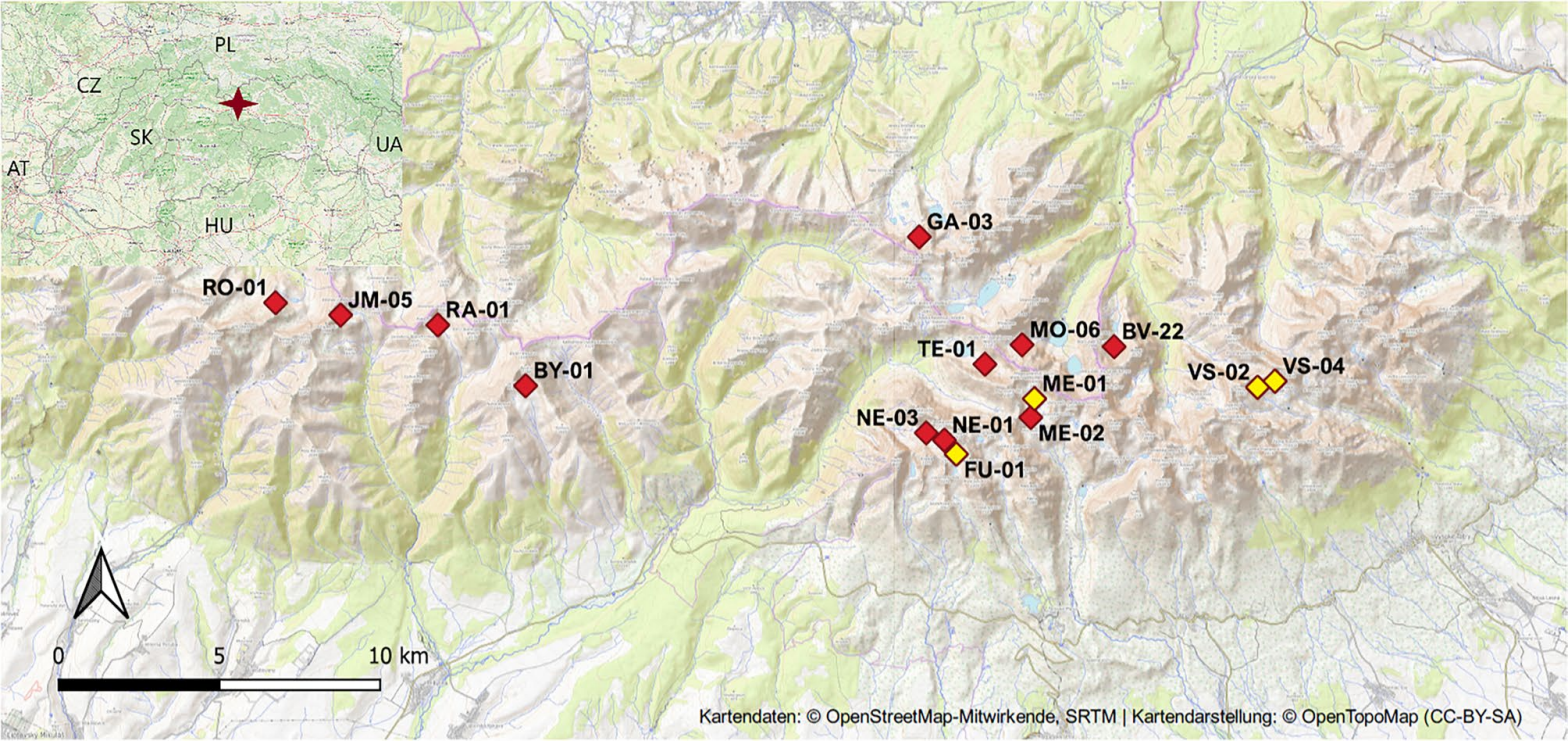
Location of sampling sites in 2015 and 2020. Full-red squares denote catchments sampled in 2015 only, red squares with yellow fill denote positions of catchments with high proportions of scree deposits analyzed in more detail in 2020 (VS-02 was sampled in 2020 only). Codes of lake catchments: BV-22 (Vyšné Žabie Bielovodské), BY-01 (Veľké Bystré), FU-01 (Vyšné Wahlenbergovo), GA-03 (Zadni Staw Gąsienicowy), JM-05 (Vyšné Jamnícke), ME-01 (Veľké Hincovo), ME-02 (Malé Hincovo), MO-06 (Wyżni Mnichowy Stawek), NE-01 (Vyšné Terianske), NE-03 (Nižné Terianské), RA-01 (Vyšné Račkové), RO-01 (Horné Roháčske), TE-01 (Veľké Temnosmrečinské), VS-02 (Pusté), and VS-04 (Ľadové). Map data came from OpenStreetMap.

The Tatra Mountains (the highest part of Carpathian chain) are situated in central Europe along the Polish-Slovak border (Fig. 1). Bedrock is granodiorite in the central part, the western part also consists of gneiss and mica schist^19^. For more details on the Tatra Mountain characteristics see Ref.^12^. The soils in alpine zone are dominated by shallow undeveloped leptosol and regosol in alpine meadows, and by sparse scree soils below surface stones in scree deposits^14^. Scree deposits investigated in this study were formed by unconsolidated bedrock material originated from physical weathering of rocks (Fig. SI-1). They were to a large extent similar to talus areas as defined by Williams et al.^20^, but were without vegetation.

Soils were sampled in the alpine zone of the Tatra Mountains (elevation of 1700–2200 m a.s.l.), where land cover is comprised mostly of scree or rocks (bare and/or covered with lichens; commonly *Rhizocarpon, Acarospora oxytona*, and *Dermatocarpon luridum*) and with sparse vegetation (dry alpine tundra dominated by *Calamagrostis villosa*, *Festuca picta*, and *Luzula luzuloides*). Two sampling surveys were conducted (Fig. 1). The first soil sampling survey aiming to compare the basic soil chemistry of scree areas (covering 5–67% of catchments^12^) and alpine meadows was performed in September 2015. The second survey was done in September 2020 and focused on comparisons of the two soil types in four catchments with the same granodiorite bedrock and with high proportions of scree deposits (> 30%). The comparison included more detailed soil analyses, including microbial biomass and its enzymatic activity.

### Soil sampling and analyses

#### The 2015 soil survey

In each catchment, one soil sample from a scree deposit and one sample from a meadow were taken.

Meadow soils were taken from 0.25 m^2^ pits (50 × 50 cm), in which two distinguishable upper horizons were excavated quantitatively. For the purpose of the study, we further use the following classification: the uppermost soil horizon rich in organic matter (A-horizon), and the underlying mineral horizon (M). Soil from each horizon was separately mixed and weighed. Scree soils were quantitatively sampled from pits (0.25–0.5 m^2^) made by the removal of stones in scree deposits to a depth of ~ 0.5 m. Fresh soil samples were then stored one week at 4 °C in the dark until further processing prior to analyses.

To remove coarse particles, soil samples were sieved through a 5-mm stainless-steel sieve. Then the < 5 mm fraction was air dried (AD) for 14–21 days between two sheets of filter paper at laboratory temperature, and finally passed through a stainless-steel < 2-mm sieve (hereafter referred as the AD soil). Before the elemental analyses, the AD soil was finely ground and homogenized. Part of the field-moist < 5 mm soil fraction was frozen at − 20 °C and stored for analyses of potential enzymatic activity.

Elements in the finely ground soil were analyzed as follows: Dry weight (DW) and loss on ignition (LOI) were obtained by drying at 105 °C for 2 h and combustion at 550 °C for 2 h in an oven, respectively. Total phosphorus (P) was determined by HNO_3_ and HClO_4_ digestion according to Ref.^21^, and total soil organic C and N contents were measured in dried (60 °C) and milled samples using an elemental analyzer (Vario MICRO cube, Elementar, Germany). The total content of metals was analysed by flame atomic absorption spectrometry (Ca, Mg, Na, K, Fe, Mn, Li, and Ti) and/or volumetric titration (Al) after the mineralization of finely ground AD soil with H_2_SO_4_, HNO_3_, and HF (200 °C, 2 h). The concentration of Si was calculated from the concentration of SiO_2_, calculated as the difference between dry weight and loss on ignition (LOI) and the concentration of metal oxides (CaO, MgO, Na_2_O, K_2_O, Al_2_O_3_, Fe_2_O_3_, MnO, Li_2_O and TiO_2_).

Oxalate-extractable Fe (Fe_ox_), Al (Al_ox_), P (P_ox_) and soluble reactive oxalate-extractable P (SRP_ox_) were determined by the extraction of 0.5 g of AD < 2 mm soil with 50 ml of acid ammonium oxalate solution (0.2 M H_2_C_2_O_4_ + 0.2 M (NH_4_)_2_C_2_O_4_ at pH 3) according to Ref.^22^, with extraction process modified by Kopáček et al.^14^. The Fe_ox_, Al_ox_, and P_ox_ concentrations were determined using the method by Kopáček et al.^21^, and SRP_ox_ colorimetrically according to Wolf and Baker^23^. Concentrations of bioavailable orthophosphates according to Olsen and Sommers^24^ (P_Olsen_) were determined after the extraction of field-moist soil with 0.5 M NaHCO_3_ (1:15; 45 min).

The effective cation exchange capacity (CEC) was calculated as the sum of exchangeable base cations (BC_EX_ = sum of Ca^2+^_EX_, Mg^2+^_EX_, Na^+^_EX_, K^+^_EX_) and exchangeable acidity (the sum of Al^3+^_EX_ and H^+^_EX_), measured by extracting of 2.5 g of dried < 2 mm AD soil with 50 ml of 1 M NH_4_Cl and 1 M KCl, respectively^14^. Concentrations of BC_EX_ were determined by ICP-MS and exchangeable acidity was determined by titration (phenolphthalein, 0.1 M NaOH) according to Thomas^25^. Base saturation (BS) was the percentage of BC_EX_ in CEC.

Concentrations of C, N, and P in the soil microbial biomass (CMB, NMB, PMB) were measured by a chloroform fumigation-extraction method^26–28^. Fresh samples (< 4 mm, 5 g), were extracted either directly or after fumigation with ethanol-free chloroform for 24 h with 40 ml of 0.5 M K_2_SO_4_ for CMB and NMB, and with 75 ml of 0.5 M NaHCO_3_ for PMB, respectively, for 45 min, and then filtrated (Whatman, No 42). The sulfate extracts were analyzed for concentrations of organic C and total N using a TOC-L analyzer equipped with a total N measuring unit (TNM-L, Shimadzu, Japan). Bicarbonate extracts were acidified with H_2_SO_4_ and analyzed for reactive P concentrations spectrophotometrically according to Brookes et al.^26^. The CMB, NMB, and PMB were calculated as differences in the concentrations of organic C, total N, and extractable P in soil extracts before and after fumigation. The values were corrected for extraction efficiencies using k_EC_ = 0.41^28^, k_EN_ = 0.45^27^, and k_EP_ = 0.4^26^.

Basal soil respiration was characterized by the rate of CO_2_ production from the soil at 10 °C. 10 g of soil in 100-ml flasks were preincubated for 10 days at 10 °C, which allowed the microbial activity to stabilize after respiratory flushes following sieving pretreatment. Then, the samples were sealed air-tight and the CO_2_ accumulation was measured after 24 h using an Agilent 6850 GC system (Agilent Technologies, Santa Clara, CA, USA). The specific respiration activity was calculated as the ratio of CO_2_-C production to CMB^29^.

Potential activities of five hydrolytic enzymes characterized the potential of microbial organic nutrient acquisition. The activity of C-mining (β-glucosidase and cellobiosidase), N-mining (Ala-aminopeptidase and chitinase), and P-mining enzymes (phosphatase) were determined using a microplate fluorometric assay^30^. Thawed soils (1 g) were mixed into 100 ml of distilled water and sonicated for 4 min; 200 μl of the soil suspension was added to 50 μl of methylumbelliferyl solution to measure the potential activities of β-glucosidase, cellobiosidase, phosphatase, and chitinase, the potential activity of Ala-aminopeptidase was measured after addition of 200 μl of the soil suspension to 50 μl of 7-aminomethyl-4-coumarin substrate solution. From pre-tested concentrations of each fluorogenic substrate (50, 100, and 300 μM), the one with the highest enzymatic activity was chosen. Plates were incubated at 20 °C for 2 h^31^, and then the fluorescence was measured with an INFINITE F200 microplate reader (TECAN, Crailsheim, Germany) at an excitation wavelength of 365 nm and an emission wavelength of 450 nm. The activities of C-, N- and P- mining enzymes, respectively, were summed and expressed as proportions (%) of the total hydrolytic activity^32^.

#### The 2020 soil survey

In 2020, soil sampling was performed to compare scree and meadow soils in four catchments with granodiorite bedrock (Fig. 1). Their respective catchment codes, areas and percentages of scree cover are as follows: Ľadové (VS-04, 13 ha; 64%), Vyšné Wahlenbergovo (FU-1, 32 ha; 46%), Pusté (VS-02, 20 ha; 43%), and Veľké Hincovo (ME-01, 127 ha; 32%). The relative proportions of scree cover was estimated using aerial geo-referenced photographs, details are given in Ref.^11^.

Meadow soils were sampled only from the upper organic-rich horizon (here called the A horizon) from pits of 0.023 m^2^ (15 × 15 cm) at nine sites within each catchment (3 samples were randomly combined by weigh to gain three representative samples per catchment). Scree soils were sampled from three representative plots in each catchment, using the same approach as in 2015.

The chemical and microbiological analyses were performed the same as in 2015, with the following exceptions: an additional determination of nutrient (C, N, P) availability by the extraction of fresh soil samples with water was performed by extracting field-moist soils with distilled water (1:10, w/v) with 1 h shaking on a horizontal shaker; the extracts were filtered (Whatman GF/C filters) and analyzed for DOC, total dissolved nitrogen (DN), NO_3_-N, NH_4_-N, and soluble reactive P (SRP_H2O_). DOC and DN were analyzed using a TOC-L analyzer equipped with the TNM-L total N measuring unit (Shimadzu, Japan). Concentrations of NO_3_-N, NH_4_-N, and SRP were determined using a flow injection analyzer (FIA Lachat QC8500, Lachat Instruments, Milwaukee, WI, USA) by the following methods: ascorbic acid reduction of phosphomolybdic acid for the analysis of SRP, a phenol-hypochlorite assay with sodium nitroprussite as the catalyst (Berthelot reaction) for NH_4_-N determination, and the diazotisation of sulfanilic acid with subsequent coupling with N-(1-naphthyl)-ethylenediamine in a strongly acid solution for NO_3_-N determination. Dissolved organic N (DON) was calculated by subtraction of NO_3_-N and NH_4_-N from DN (DON = DN–NH_4_–N–NO_3_–N).

The isotopic composition of soil C was characterized using an NC Elemental analyzer (ThermoQuest, Germany) connected to an isotope ratio mass spectrometer (IR-MS Delta X Plus, Finnigan, Germany). The measured ratio of ^13^C/^12^C was expressed as δ^13^C using a Vienna PDB standard.

All data on chemistry and biochemistry in this study are given as related to the DW (105 °C) fraction.

Due to a non-normal data distribution as tested by the Shapiro–Wilk test (*p* < 0.01), the non-parametric Mann–Whitney U was chosen for testing differences between soil types. All statistical tests were performed using XLSTAT 2022 software (Addinsoft).

## Results

### Basic soil characteristics

Soils in alpine meadows were generally shallow. The upper organic-rich horizon A was on average 14 cm deep (5–36 cm), densely rooted and rich in stones (the average ratio of removed coarse stones >  ~ 5 cm to unsieved soil ratio was 1.4 on site). In places with deeper soils, the A horizon gradually turned into the lighter colored M horizon. The amount of soils (dry weight < 2 mm fraction) varied between 8–85 kg m^−2^ in the A horizon, and 0–125 kg m^−2^ in the M horizon (Table 1). In the 2020 soil survey, we observed an average depth of the A horizon of 11 cm, underlain by regolith (Table 2).

**Table 1:** Basic chemical characteristics of alpine meadow (horizons A and M), and scree soils sampled in 2015 from 14 Tatra Mountains catchments (minimum–maximum values with medians in parentheses).

		Meadow A	Meadow M	Scree
		n = 14	n = 9	n = 14
Depth	cm	5–36 (14)	0–36 (21^†^)	n.m
Soil (< 2 mm)	kg m^−2^	8–85 (32)	0–125 (71^†^)	4–16 (9)^††^
pH_H2O_		4.24–4.88 (4.63)	4.58–5.1 (4.81)	4.39–4.88 (4.61)
pH_CaCl2_		3.35–4.10 (3.77)	3.66–4.23 (4.0)	3.57–4.05 (3.79)
LOI	%	13–41 (23)^b^	6–18 (11)^a^	5.8–17.5 (11.1)^a^
C	mmol g^−1^	6.2–18.2 (9.6)^b^	2.8–8.2 (4)^a^	2.2–8.2 (5)^a^
N	mmol g^−1^	0.34–1.04 (0.6)^c^	0.16–0.48 (0.19)^a^	0.12–0.44 (0.32)^b^
P	µmol g^−1^	17.4–59 (32.2)^b^	10.7–45.9 (21.1)^a^	18.2–39.43 (27.3)^ab^
C:P	molar ratio	64–222 (311)^b^	135–387 (224)^a^	79–266 (197)^a^
C:N	molar ratio	13.4–20 (16.5)^a^	14.7–24.1 (19.2)^b^	13.9–18.6 (16.4)^a^
P_ox_	µmol g^−1^	8.2–26 (14.2)^ab^	5.2–29.1 (10.8)^a^	8.4–22 (15.4)^b^
SRP_ox_	µmol g^−1^	3–8.8 (4.1)^a^	1.4–8.8 (5.7)^a^	5.3–16.3 (10)^b^
Al_ox_	µmol g^−1^	47–345 (151)^b^	88–377 (209)^b^	42–159 (86)^a^
Fe_ox_	µmol g^−1^	24–148 (83)	24–213 (96)	42–103 (61)
(Al + Fe)_ox_	µmol g^−1^	91–492 (223)^b^	112–509 (309)^b^	85–219 (147)^a^
P_Olsen_	µmol g^−1^	0.1–0.3 (0.17)^a^	0.04–0.34 (0.18)^a^	0.16–3.5 (1.95)^b^
Na^+^_EX_	µeq g^−1^	0.2–1.3 (0.6)^b^	0.1–1 (0.3)^a^	0.1–0.3 (0.2)^a^
K^+^_EX_	µeq g^−1^	2.2–9.1 (4.9)^b^	0.4–2.4 (1.3)^a^	0.7–2.0 (1.3)^a^
Mg^2+^_EX_	µeq g^−1^	1.4–7.1 (4.0)^b^	0.3–1.8 (0.7)^a^	0.3–2.1 (0.9)^a^
Ca^2+^_EX_	µeq g^−1^	2.1–18 (6.3)^b^	0.4–4.3 (2.6)^a^	1.1–5.3 (1.8)^a^
H^+^_EX_	µeq g^−1^	8.5–77 (22.5)^b^	2.4–20.7 (14.5)^a^	3.3–21.3 (10.7)^a^
Al^3+^_EX_	µeq g^−1^	72–152 (99)^b^	49–123 (94)^b^	31–89 (66)^a^
BC_EX_	µeq g^−1^	6.6–95 (15)^b^	1.7–9.2 (4.4)^a^	2.3–8.4 (4.4)^a^
BS	%	5–45 (12.6)^a^	1.2–13 (5.3)^a^	3.6–20 (5.9)^a^
CEC	µeq g^−1^	107–212 (152)^c^	55–145 (110)^b^	43–109 (79)^a^

*n.m.* not measured.

Different upper-case letters indicate statistically significantly (*p* < 0.05) different chemical parameters.

^†^The median was calculated only for cases if the M horizon was present.

^††^Up to 0.5 m under the surface.

**Table 2:** Basic chemical characteristics of alpine meadow (A horizon) and scree soils sampled in 2020 in 4 selected Tatra Mountain catchments with a high proportion of scree deposits (Fig. 1).

		Meadow	Scree
		n = 12	n = 12
pH_H2O_		4.12–4.73 (4.38)	4.24–4.16 (4.46)
pH_CaCl2_		3.48–3.91 (3.69)	3.73–4.13 (3.87)**
LOI	%	4.9–43 (15.4)	4.3–11.8 (6.2)**
C	mmol g^−1^	1.6–17.9 (6)	1.2–4.7 (2.6)**
N	mmol g^−1^	0.11–1.1 (0.39)	0.09–0.28 (0.17)**
P	µmol g^−1^	18–52 (26)	11–32 (21.8)*
P_ox_	µmol g^−1^	8–28.2 (17.6)	5.2–21.1 (16.1)
SRP_ox_	µmol g^−1^	2.1–4.4 (3)	3.5–16.3 (10)***
Al_ox_	µmol g^−1^	57–210 (163)	48–203 (87)**
Fe_ox_	µmol g^−1^	27–101 (58)	34–87 (67)
(Al + Fe)_ox_	µmol g^−1^	102–275 (228)	68–213 (119)*
P_Olsen_	µmol g^−1^	0.02–0.28 (0.11)	0.5–2.4 (1.5)***
C:P	Molar ratio	0.05–0.34 (0.24)	0.06–0.29 (0.12)*
C:N	Molar ratio	13.2–18.6 (15.9)	12.8–16.9 (15.4)
Na^+^_EX_	µeq g^−1^	0.3–1 (0.8)	0.12–0.48 (0.3)***
K^+^_EX_	µeq g^−1^	2.3–8.4 (3.9)	0–1.3 (0.7)***
Mg^2+^_EX_	µeq g^−1^	2.1–8.2 (4)	0.3–0.9 (0.6)***
Ca^2+^_EX_	µeq g^−1^	2.4–21.1 (5.9)	0.5–3.4 (1)***
H^+^_EX_	µeq g^−1^	15.6–54 (28)	9.2–18 (13)***
Al^3+^_EX_	µeq g^−1^	62–113 (88)	30–60 (49)***
BC_EX_	µeq g^−1^	8.4–34 (15.2)	1.5–4.1 (2.6)***
BS	%	6.7–23 (10.7)	2.2–9.1 (4.1)***
CEC	µeq g^−1^	95–178 (138)	43–79 (65)***

Ranges and medians (in brackets) are given.

Asterisks denote significant differences: *p < 0.05, **p < 0.01, ***p < 0.001.

The diameter of stones in scree deposits varied from centimeters to meters. Scree soils below the upper layer of stones were deposited in gaps between stones or on surfaces of deeper stones (Fig. SI-1). Hence, the scree soils did not form a continuous layer, but appeared in isolated deposits weighing from grams to kilograms. The estimated amount of soil (< 2 mm fraction; dry weight) ranged between 4 and 16 kg m^−2^, with an average of 9 kg m^−2^ in the 0.5 m upper layer of scree. Similar scree soil amounts were also observed in the 2020 survey, with an average of 9 kg m^−2^ in the upper 0.5 m scree layer, varying from 4 in the VS-02 to 14 kg m^−2^ in the FU-01 catchment; details are given in Supplementary information (Table SI-1).

### Soil chemistry

**Figure 2:**
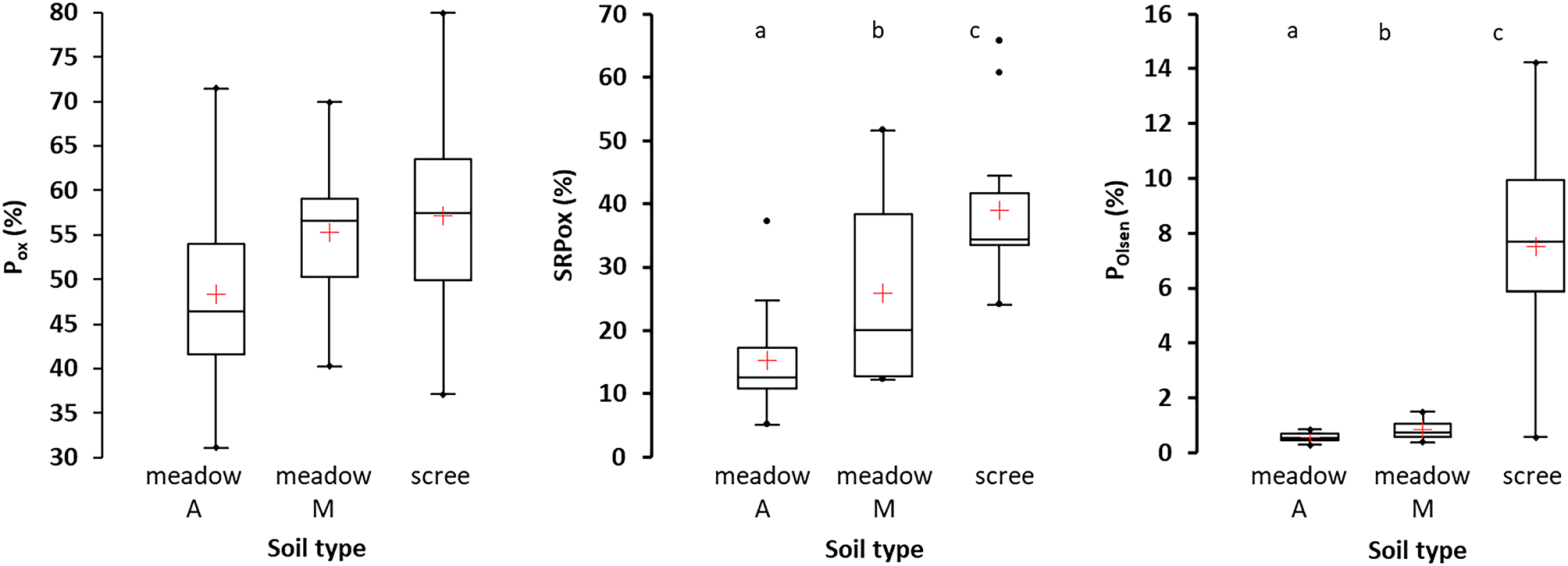
Comparison of the proportion (%) of extractable P forms in total P in alpine meadow soils (horizons A and M), and scree soils. P_ox_—oxalate-extractable P, SRP_ox_—oxalate-extractable soluble reactive P, P_Olsen_—P measured in HCO_3_^-^ extract according to Olsen and Sommers^24^. Ranges, outliers, and 25% and 75% quartiles are given, horizontal lines within the boxes denote medians, red crosses denote averages. Different letters denote statistically significant differences.

All the soils were acidic, with pH_H2O_ ranging between 4.1–5.1 and pH_CaCl2_ between 3.35–4.1 (Tables 1, 2), with no significant difference between meadow and scree soils. The chemical composition of scree soils was similar to soils in the M horizon of alpine meadows. Both these soil types contained less C and BC_EX_ than the meadow A horizon (Table 2). However, there were several important differences between the scree and M horizon soils. Scree soils had lower concentrations of total N, Al_ox_, Al^3+^_EX_, and CEC than the M horizons, but higher concentrations of all extractable P forms (P_ox_, SRP_ox_, P_Olsen_) (Table 1), and also higher proportions of SRP_ox_ and P_Olsen_ in total P concentrations (Fig. 2).

Total concentrations of metals and Si were generally similar in the M horizons of scree and meadow soils (Table SI-2). The main significant differences between these soil types were the higher total concentrations of Ca, Na, and Mn in scree soils than in the meadow M horizon (for more details see Table SI-2).

The 2020 soil survey in the four selected catchments confirmed the general pattern of differences between meadow and scree soils observed in 2015 (Table 2; details for individual catchments are given in Table SI-3). Scree soils significantly differed from meadow soils in the A horizon in concentrations of P species. Scree soils exhibited markedly higher concentrations of extractable P forms compared to meadow soils despite their slightly lower content of total P (21.8 *vs.* 26 µmol g^−1^; *p* < 0.05) and no significant differences in P_ox_ concentrations. The SRP_ox_ concentrations were three times higher in the scree than meadow soils (10 *vs.* 3 µmol g^−1^; *p* < 0.001), and their P_Olsen_ concentrations were even one order of magnitude higher (1.5 *vs.* 0.11 µmol g^−1^; *p* < 0.001).

The concentrations of P_ox_ were significantly correlated with concentrations of Al_ox_ and Fe_ox_, with stronger relationships in scree than in meadow soils. Scree soils had similar Fe_ox_ concentrations to meadow soils (58 and 67 µmol g^−1^) but lower Al_ox_ contents (87 *vs.* 163 µmol g^−1^; *p* < 0. 01).

**Figure 3:**
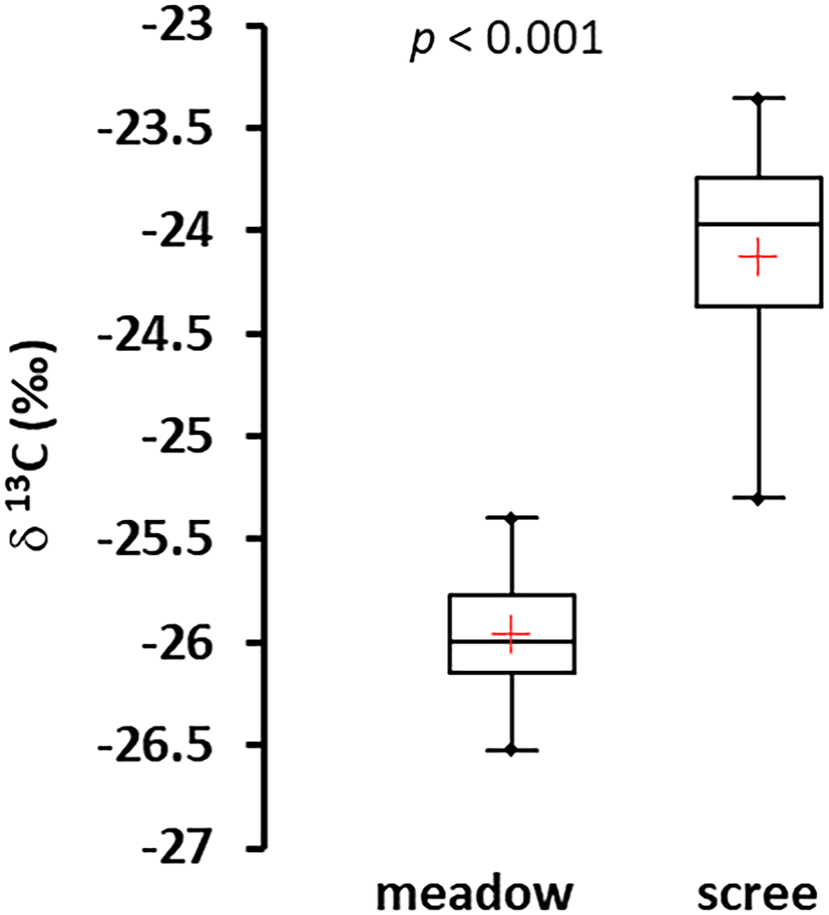
Comparison of the isotopic composition of soil C in alpine meadow soils (horizon A) and scree soils from 4 catchments sampled in 2020 (FU-1, ME-01, VS-02, VS-04). In each catchment, three soil samples for each of both soil types were analyzed. Ranges and 25% and 75% quartiles are given, horizontal lines within the boxes denote medians, and red crosses denote averages.

The meadow soils had higher total C contents than scree soils, and a lower δ^13^C (− 25.96‰ compared to − 24.12 ‰ in scree soils, Fig. 3) indicating different origins of organic matter for these soil types.

**Figure 4:**
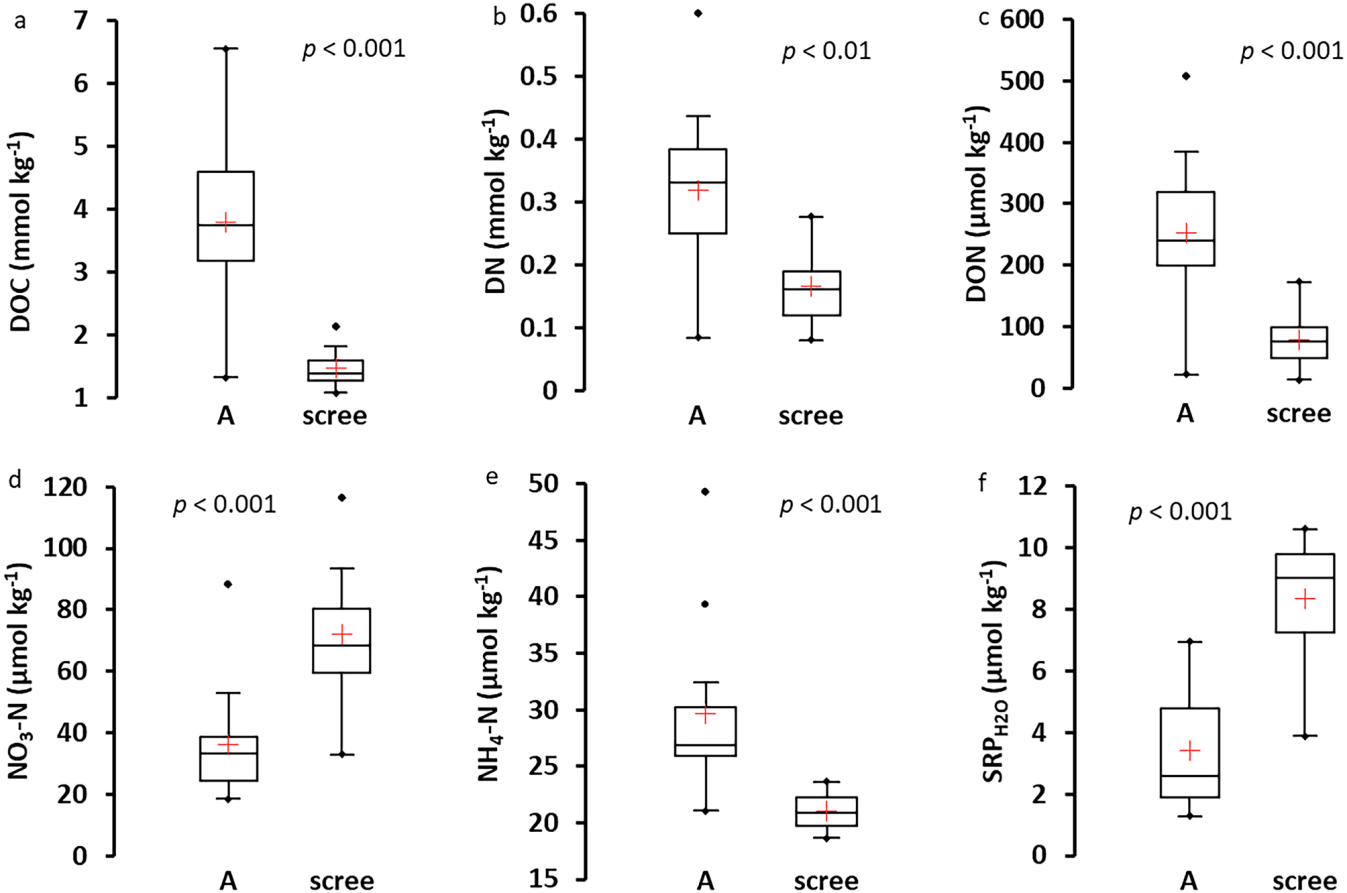
Concentrations of water-extractable forms of C, N, and P in alpine meadow soils (horizons A) and scree soils sampled in four scree-rich catchments in 2020. Ranges, outliers, and 25% and 75% quartiles are given, horizontal lines within the boxes denote medians, and red crosses denote averages.

Concentrations of water-extractable C, N, and P forms also differed between the soil types. The meadow soils had higher concentrations of DOC, DN, DON, and NH_4_-N, but lower NO_3_-N (72 *vs.* 36 µmol kg^−1^; *p* < 0.001) and SRP_H2O_ concentrations (8.3 *vs.* 3.4 µmol kg^−1^; *p* < 0.001) than scree deposit soils (Fig. 4).

### Soil microbial and biochemical parameters

The microbial biomass in scree soils was similar to that in the M horizon in meadows, but substantially lower compared to the meadow A horizon (Table 3). The CMB and NMB concentrations were about 5 times lower in scree soils than in the meadow A horizon (34 *vs.* 168 µmol g^−1^ and 2.8 *vs.* 15.4 µmol g^−1^, respectively), and the PMB concentration was ~ 3 times lower (1.7 *vs.* 5.6 µmol kg^−1^; *p* < 0.001). The detailed 2020 soil survey thus confirmed that scree soils contained a lower microbial biomass than meadow soils (Table 3).

**Table 3:** Microbial characteristics of alpine meadow and scree deposit soils sampled in 2015 from 14 catchments, and in 2020 from 4 catchments in the Tatra Mountains (data show ranges, averages are in parenthesis).

		The 2015 soil survey			The 2020 soil survey	
		A horizon (n = 14)	M horizon (n = 9)	Scree soil (n = 14)	A horizon (n = 12)	Scree soil (n = 12)
CMB	µmol g^−1^	46–480 (168)^b^	16–66 (33)^a^	19–51 (34)^a^	39–476 (185)	7–21 (14)***
NMB	µmol g^−1^	4–42 (15.4)^b^	0.9–8.2 (3.0)^a^	0.8–5.5 (2.8)^a^	4–54 (23)	1.3–4 (2.6)***
PMB	µmol g^−1^	1.8–13.5 (5.6)^b^	0.3–2.5 (1.2)^a^	0.3–4.3 (1.7)^a^	1.1–18.3 (5.7)	0.2–1.3 (0.7)***
Resp.	µmol C g^−1^ d^−1^	0.53–1.7 (1.04)^b^	0.03–0.3 (0.2)^a^	0.13–0.46 (0.3)^a^	0.6–2.4 (1.3)	0.07–0.29 (0.15)***
CO_2_-C:CMB	µmol mol^−1^	3.2–12.4 (7.2)	1–10.2 (6.8)	4.7–11.3 (8.6)	5–16 (8.1)	4.9–26.5 (11)*

*CMB and NMB* C and N in the microbial biomass, respectively. *Resp.* respiration (CO_2_-C production), *CO*_*2*_*-C* CMB specific respiration.

Different letters in the 2015 survey section denote statistically significantly different values.

In the 2020 survey section, asterisks indicate statistically significant differences: *p < 0.05, **p < 0.01, ***p < 0.001.

**Figure 5:**
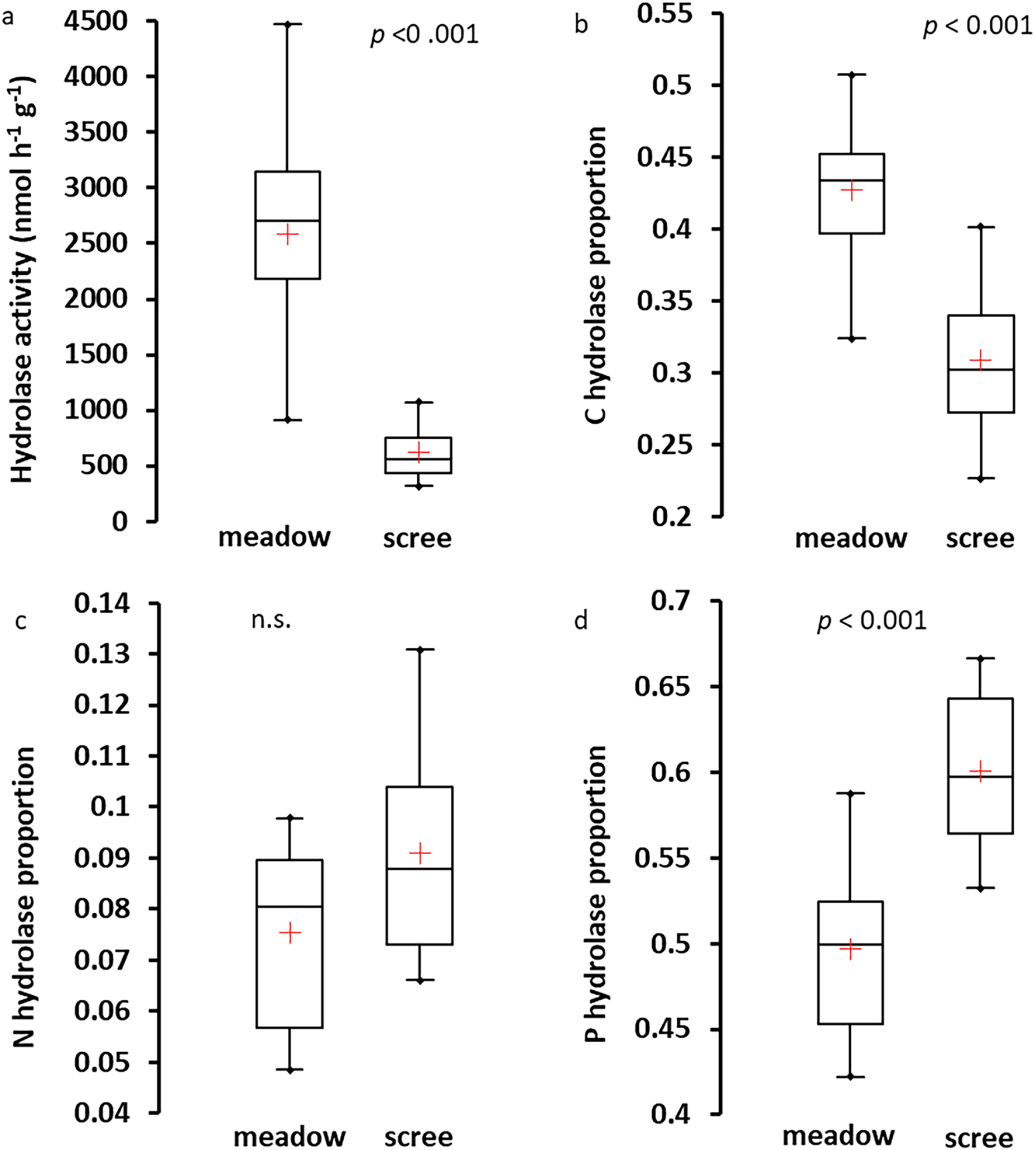
Comparisons of total hydrolytic activity (**a**) and relative enzymatic investments into C-mining (**b**), N-mining (**c**), and P mining (**d**) within the total activity of hydrolases in meadow alpine soils (A horizon) and scree soils in four selected Tatra Mountain catchments (Fig. 1) in 2020. Ranges, and 25% and 75% quartiles are given, horizontal lines within the boxes denote medians, and red crosses denote averages.

In concert with their lower microbial biomass, scree soils exhibited almost one order of magnitude lower respiration rates than soils in the A horizon of meadows (0.15 *vs.* 1.3 µmol C g^−1^ d^−1^) and a lower total activity of hydrolytic enzymes (Fig. 5). However, microbial communities in scree soils were more active compared to those in meadow soils, evidenced by their higher biomass-specific respiration rate (CO_2_-C:CMB of 11 *vs.* 8 µmol mol^−1^; *p* < 0.05) and enzymatic activity. The relative enzymatic investments into P-gaining was higher but that into C-gaining lower in scree soils than in meadow soils (Fig. 5).

## Discussion

To assess the role of scree deposit soils in the biogeochemistry of alpine catchments, and to gain insights into their role in terrestrial P cycling and supply to lakes, we estimated the amounts and properties of scree soils in the alpine zone of the Tatra Mountains. We found 4–16 kg m^−2^ of scree soils in 14 alpine catchments in 2015, as well as in 4 scree-rich catchments in 2020. These scree soil pools were similar to previous estimates^14^ which reported on average 13 kg m^−2^ of dry weight < 2 mm soils in the upper 0.5 m layer of scree areas of six Tatra Mountain catchments. We are aware that these values probably underestimate the total amount of scree soils due to the relatively shallow sampling resulting from the physical impossibility of reaching deeper levels by manual removing stones (no mechanization or terrain disturbance is allowed in the protected areas). Hence, we suppose that the total amount of soil accumulated in the whole profile of scree areas is likely higher than reported here for the uppermost 0.5 m. Our estimates should thus be considered as a lower limit for scree soils in the Tatra Mountain catchments.

The comparison of scree and meadow soils revealed general similarities in their basic elemental composition, reflecting their common origin in the physico-chemical weathering of parent granodiorite bedrock. Both soils are relatively rich in P, which is, however, present primarily in forms inaccessible for organisms. On the other hand, these soil types largely differ in microbial and biochemical parameters, primarily connected with inputs of organic matter having different quantity, quality and origin.

Alpine meadow soils are substantially affected by plant activity. The presence of higher plants is connected with the input of organic matter via litter and rhizodeposition, and its transformation mediated by the activity of soil microorganisms^33^. The accumulation of soil organic matter leads to the formation of an A horizon, whose chemistry differs from the deeper mineral M horizon as well as from scree soils. Larger contents of organic C, N, and P in meadow A horizons than in scree soils is connected with a larger microbial biomass, which is evidenced by higher rates of exoenzymatic activities, and a higher C mineralization rate. Larger relative investments into C-mining enzymes in meadow compared to scree soils (Fig. 5b) indicate that microbial metabolism targets the decomposition of complex C-rich organic compounds of plant origin. The high biological activity in the rhizosphere, consisting of root and microbial respiration and organic acid production, likely also intensified chemical weathering^34,35^. This enriched the meadow soil with extractable forms of base cations in the A horizon, and increased Al_ox_ and Fe_ox_ concentrations^36^ in both A and M horizons (Table 2) compared to the scree soil, which enhanced the potential of P retention in alpine meadow soils^37^. The high biological activity within the organic layer also influenced the mineral horizons of meadow soils. Intensified weathering and element leaching with organic acids during pedogenesis probably decreased the contents of total Ca and Na in the M horizons of meadow soils in comparison to the scree soils, which were otherwise similar in many parameters (Table 2, Table SI-2).

In contrast to meadow soils, the effect of vegetation is absent during the development of scree soils. They contained organic C in similarly low amounts as the M horizons of meadow soils (< 10 mol kg^−1^). The C concentration in scree soils was markedly enriched in ^13^C compared to the isotopic composition of plant-derived C in meadow soils (Fig. 3), indicating a different origin. The organic matter in scree soils probably originates mainly from the activity of prokaryotic autotrophs (cyanobacteria and other autotrophic bacteria, e.g., nitrifiers), which have a higher δ^13^C signature of bulk biomass compared to C3 plants^38,39^ (Alexander et al. 2006; Wada et al. 2012) with a minor potential contribution of allochtonous organic C sources like lichens, mosses, and wind-delivered organic litter and dust, including snow dust as described by^40^ Ley et al. (2004). Prokaryotes likely dominate the microbial communities in scree soils. A similar situation was observed in thin “soils” of non-vegetated areas in the low Arctic^41^ in habitats similar to the scree soils in alpine regions. The dominance of bacteria in the microbial community of scree soils could also explain the lower biomass C:N ratio (Table SI-3) and higher biomass-specific enzymatic and respiratory activity compared to meadow soils (Table 3). The low proportion of hydrolytic activity targeted to C-mining (Fig. 5b) is in accord with the likely high degradability (due to the absence of plant structural polymers) of organic matter of microbial origin. In addition, the investment into N-mining in both soil types was very low (Fig. 5c), which can be explained by the N saturation of thin soils in alpine catchments due to the high long-term atmospheric N deposition in the Tatra Mountains^1^. In contrast, the high proportion of phosphatase within the hydrolytic activity in scree soils (~ 60% of the total hydrolytic activity, Fig. 5d), was significantly higher than in meadow soils, pointing to targeted fast “P-mining” from organic matter. We suggest that these unique properties of scree soils (i.e., the high biomass-specific activity of the microbial community connected with rapid turnover of the small pool of soil organic matter, and targeted P-mining from this pool) may contribute to a higher potential for P leaching from scree than meadow soils.

The differences in soil chemistry also pointed to a higher P mobility in scree soils compared to meadow soils. Scree soils contained more than two-times higher amounts of the most mobile P form (i.e., SRP_H2O_), indicating a higher potential for P loss. As shown^10^, the concentrations of SRP_H2O_ extracted from the Tatra Mountain soils increase when soil water pH increases from 3 to 3.5 to higher values. However, even at the similarly low pH range in both meadow and scree soils, the SRP_H2O_ release was about four-times higher from scree soils^10^. The present pH values are > 3.5 (Table 3) and significantly higher in scree soils, which favors the release of P and further enhances the potential for P losses. Scree soils further showed a higher ratio of mobile SRP_ox_ to P_ox_ than meadow soils. Concentrations of Al_ox_ and Fe_ox_ were lower in scree than in meadow soils (Table 3), which is in concert with their about two-times lower ability to retain phosphate^10^. A higher potential for P loss from scree soils is also indicated by the order of magnitude higher P_Olsen_ concentrations. In contrast to meadow soils, concentrations of P_Olsen_ in scree soils were well above the threshold value of 20 mg kg^−1^ (0.65 µmol g^−1^) above which the release of P from soils during rainfall has been suggested to increase^42^. Because the majority of scree deposits reaches the banks of the study lakes, there are absent meadow soil areas potentially able to reduce the direct nutrient fluxes from the scree slopes to lakes.

Scree soils thus had a lower potential than alpine meadows to immobilize P in organic matter due to the absence of plants and lower microbial biomass, but also due to their lower P retention capacity related to their lower Al_ox_ and Fe_ox_ concentrations^10,37^, and had higher concentrations of mobile P forms. Leaching of P from soils to waters is therefore more probable in scree than meadow soils. P concentrations have been recently increasing in some high elevation Tatra Mountain lakes, which typically have catchments with large proportions of scree deposits^12^. All four catchments studied in 2020 belonged to the group of rocky or meadow-rocky catchments, representing 25 from 30 long-term studied alpine catchments in the Tatra Mountains^5^. In these scree-rich catchments, higher annual increases in the in-lake total P (and chlorophyll *a*) concentrations were documented from 1992 to 2018 compared to catchments with higher meadow proportions^12^. For example, concentrations of total P in four lakes belonging to the group of scree-rich catchments increased from 0.05 to 0.08 µmol L^−1^ in 2000 to 0.07–0.14 µmol L^−1^ in 2014^10^. Because atmospheric P inputs are stable in this area^15^, this increase is probably associated with elevated P losses from the catchments. This may be associated with either an elevated terrestrial P source or a decreasing ability of soils to immobilize deposited P. A climate-driven increase in the physical weathering of rocks, which is more pronounced in scree deposits than in alpine meadows with rocks insulated by soil^12^ may also provide additional P sources. Physical (followed by chemical) weathering of unstable scree rocks could be accelerated by increasing frequency of heavy rains, number of days without snow cover, and number of days with temperature fluctuations around zero (i.e., the number of freeze–thaw cycles), which were documented in the Tatra Mountains during the last 30 years^12^. Decrease in the ability of soils to retain P is associated with their recovery from atmospheric acidification, and this process is more pronounced for scree than alpine soils^10^. In addition, we further show that soil microbial processes are able to mobilize more P available for leaching in scree soils, while more effectively immobilizing P in alpine meadows. All three processes probably contribute to the recent more rapid P loading of lakes in catchments with a large proportion of scree than in catchments dominated by alpine meadows.

## Conclusions

We characterized the chemical properties of scree soils, a relatively unknown aspect of alpine catchment ecosystems. We compared their biochemical and chemical parameters with meadow soils in alpine catchments in the Tatra Mountains, focusing on their potential to release P. Scree deposit soils had a lower microbial biomass with a higher biomass-specific activity, and a higher proportion of P-mining enzymes compared to meadow soils. The chemical and microbial composition of meadow soils is affected by large inputs of plant-derived complex organic matter, transformed by large microbial communities with hydrolytic activity targeting C-mining. These differences in the microbial activity and biochemical processes of scree and meadow soils affect their ability to retain/release P. Scree deposit soils exhibited higher concentrations of mobile P forms (SRP_ox_, P_Olsen_, SRP_H2O_), and lower concentrations of Al_ox_ and Fe_ox_, which together with higher pH, low organic matter and a low microbial biomass, led to a lower ability of scree soils to retain P. The leaching of P from scree deposit soils may thus represent an important P source for alpine lakes. The combination of climate change, recovery from atmospheric acidification, and specific characteristics of scree areas and their soils is probably responsible for the increasing input of P to alpine lakes in the Tatra Mountains. Similar changes also could be expected in other alpine areas recovering from acidification, especially in lakes with scree-rich catchments.

### Supplementary Information

Supplementary Information.

## Data Availability

The data can be provided by authors after request. Data on soil chemistry by Jiří Kaňa (jiri.kana@centrum.czjiri.kana@centrum.cz), data on soil biochemistry by Eva Kaštovská (ekastovska@prf.ju.cz).
