# Supplementary material for: Undeveloped till soils in scree areas are an overlooked important phosphorus source for waters in alpine catchments
Source: Sci Rep. 2023 Sep 7;13:14725. doi: 10.1038/s41598-023-42013-4 (PMC10485049; doi:10.1038/s41598-023-42013-4)
Supplement: Supplementary file 1 — Supplementary Information. [file 41598_2023_42013_MOESM1_ESM.docx]

**Undeveloped till soils in scree areas are an overlooked important phosphorus source for waters in alpine catchments**

Jiří Kaňa, Eva Kaštovská, Michal Choma, Petr Čapek, Karolina Tahovská, Jiří Kopáček

(SI includes 2 pages with 1 figure, 3 tables, and 1 citation)

**
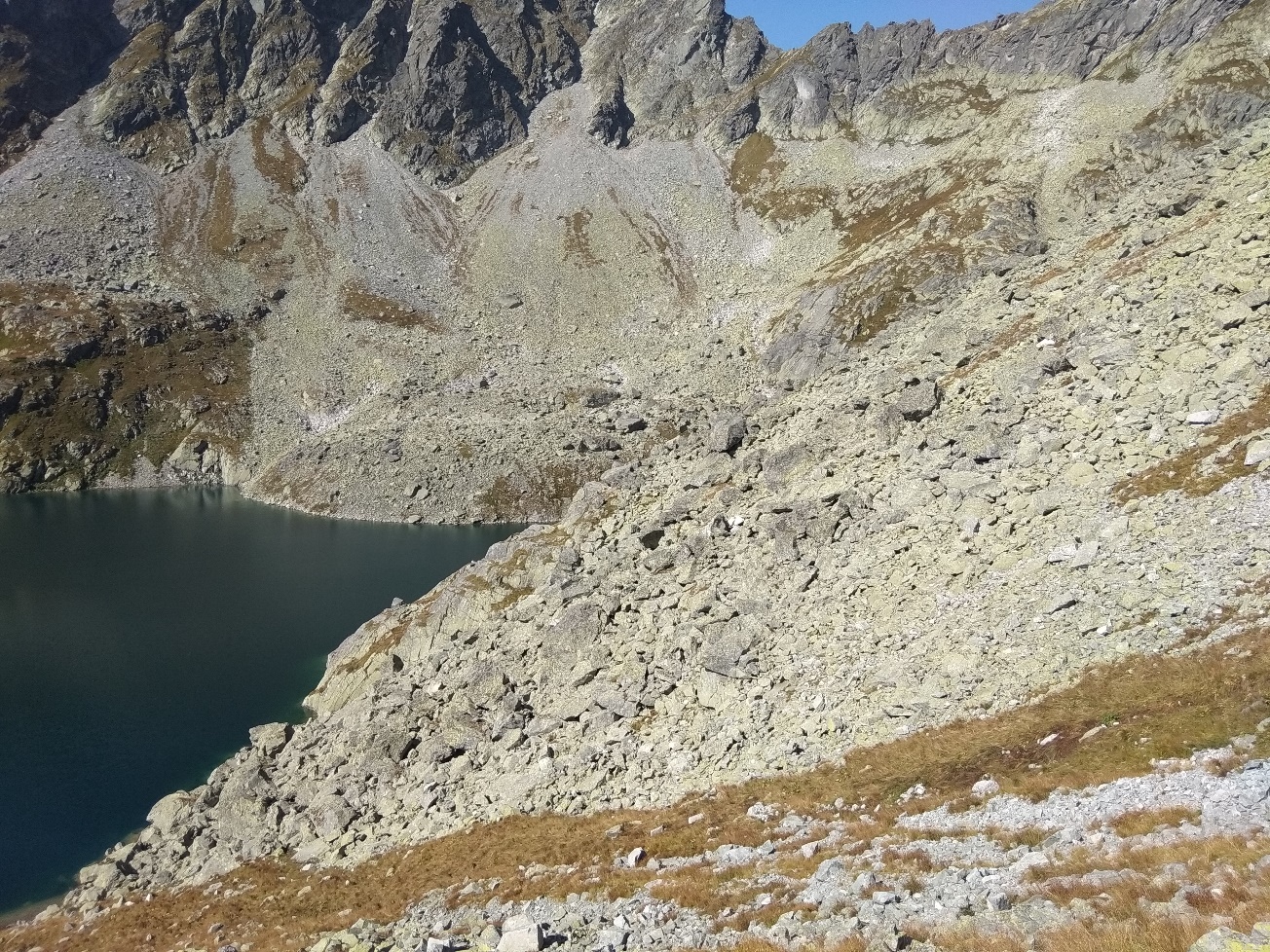

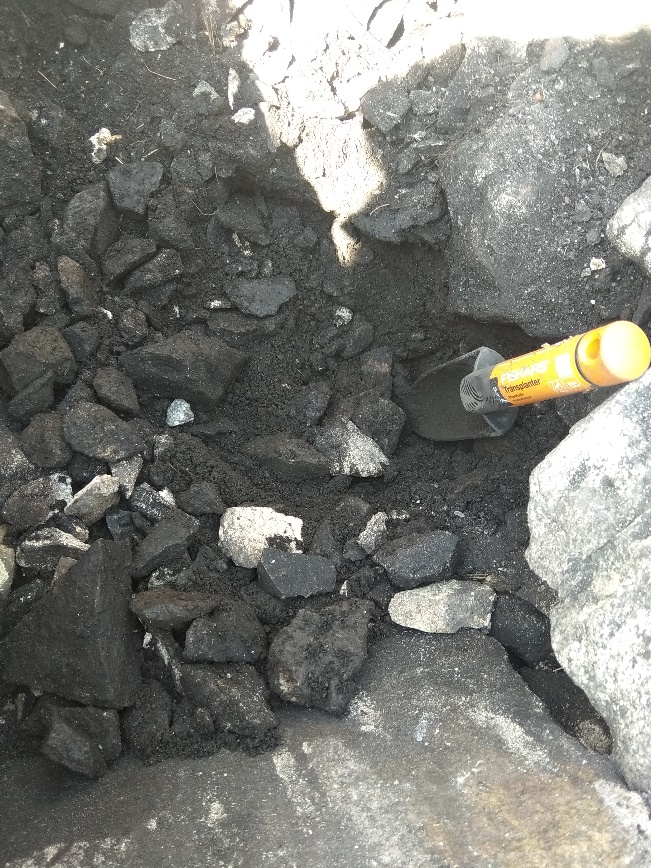

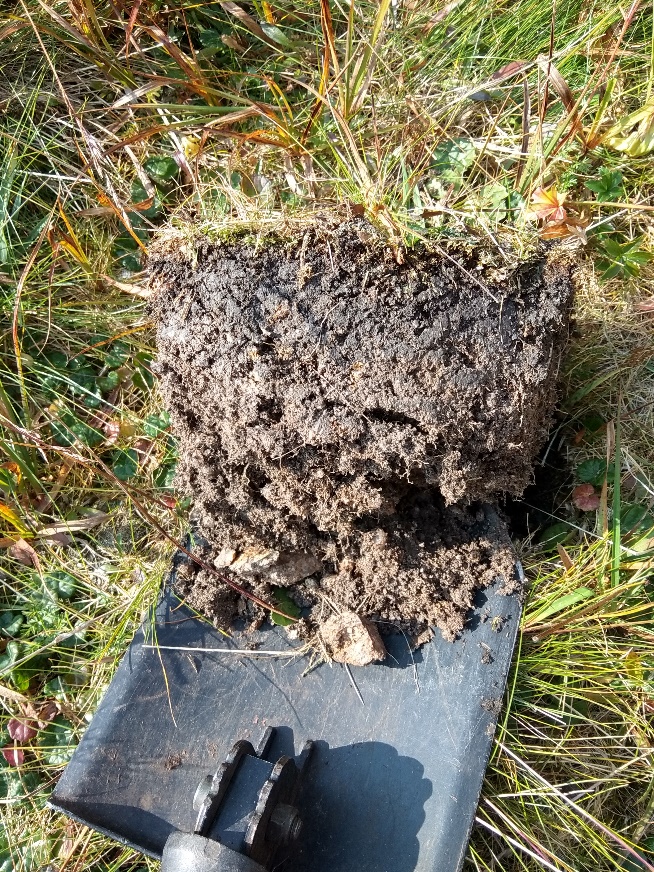
**

**c**

**b**

**a**

**FIG. SI-1** A part of alpine catchment with scree deposits and patchy meadows (a). Scree deposit soil (b), and meadow soil (c). Photo: J. Kaňa

**Table SI-1** Parameters of alpine lake catchments sampled in soil survey in 2020. Data are from Kopáček et al. (2006)

|  | Altitude of lake | Catchment | Max. relief | Scree | Meadow | Scree soil | Meadow soil |
| --- | --- | --- | --- | --- | --- | --- | --- |
|  | m | ha | m | Area proportion^†^ | | <2 mm | <2 mm |
|  |  |  |  | % | | kg m^-2^ | kg m^-2^ |
| Vyšné Wahenbergovo | 2151 | 32 | 775 | 46 | 4 | 14 | 24 |
| Veľké Hincovo | 1945 | 127 | 486 | 32 | 29 | 11 | 11 |
| Pusté | 2056 | 20,4 | 328 | 43 | 40 | 4 | 13 |
| Ľadové | 2057 | 13 | 293 | 64 | 28 | 7,5 | 10 |

^†^The rest to 100% are bare rocks.

**Table SI-2** Total content of Si and metals in soils from alpine meadows and scree deposits in catchments of 14 Tatra Mountain lakes, sampled in 2015. Ranges and averages (in brackets) are given. Different letters denote statistically significantly different values

|  |  | Meadow A | Meadow M | Scree |
| --- | --- | --- | --- | --- |
|  |  | n = 14 | n = 9 | n = 14 |
| **Si** | mol kg^-1^ | 6.2–10­.3 (8.5)^a^ | 8.7–11­.1 (10.1)^b^ | 8.9–10­.8 (10)^b^ |
| **Ti** | mmol kg^-1^ | 34–96 (50) | 26–125 (60) | 38–74 (47) |
| **Al** | mol kg^-1^ | 1.8–2.9 (2.4)^a^ | 2.7–3.3 (2.9)^b^ | 2.3–3.4 (2.9)^b^ |
| **Fe** | mol kg^-1^ | 0.16–0.42 (0.28)^a^ | 0.21–0.45 (0.31)^ab^ | 0.25–0.48 (0.32)^b^ |
| **Mg** | mol kg^-1^ | 0.11–0.22 (0.16)^a^ | 0.12–0.25 (0.18)^ab^ | 0.15–0.27 (0.2)^b^ |
| **Mn** | mmol kg^-1^ | 1.4–4.4 (2.9)^a^ | 1.7–7.5 (3.7)^a^ | 2.1–11.3 (3.9)^b^ |
| **Ca** | mmol kg^-1^ | 0.02–0.25 (0.09)^a^ | 0.01–0.23 (0.09)^a^ | 0.03–0.27 (0.15)^b^ |
| **Li** | mmol kg^-1^ | 2–4.7 (2.9)^a^ | 2.7–5.4 (3.6)^b^ | 2–6 (3.7)^b^ |
| **Na** | mol kg^-1^ | 0.35–0.85 (0.63)^a^ | 0.41–1.2 (0.77)^b^ | 0.58–1.2 (0.97)^c^ |
| **K** | mol kg^-1^ | 0.33–0.67 (0.48)^a^ | 0.44–1 (0.62)^b^ | 0.44–0.79 (0.58)^b^ |

**Table SI-3** Particular soil phosphorus forms, microbial biomass and δ 13C in 4 alpine lake catchments sampled in 2020 – a detailed insight into differences between catchments. Averages ± standard deviations are given. TP – total P, P_ox_ and SRP_ox_ – total and soluble reactive P in oxalate extract, P_Olsen_ – Olsen P, SRP_H2O_ – soluble reactive P in H_2_O extract from field moist fresh soil, CMB, PMB, NMB – C, P, and N in microbial biomass.

|  |  | Vyšné Wahenbergovo | | Veľké Hincovo | | Pusté | | Ľadové | |
| --- | --- | --- | --- | --- | --- | --- | --- | --- | --- |
|  | code | FU-01 | | ME-01 | | VS-02 | | VS-04 | |
|  |  | A | scree | A | scree | A | scree | A | scree |
| TP | mmol kg^-1^ | 20.5 ± 4.7 | 20.1 ± 4.6 | 27 ± 3 | 25.9 ± 5.2 | 23.8 ± 5.5 | 15.6 ± 5.9 | 34.4 ± 14.1 | 20.7 ± 1.7 |
| P_ox_ | mmol kg^-1^ | 14 ± 5.8 | 13.8 ± 4.6 | 16.1 ± 1.7 | 19.8 ± 0.8 | 18.7 ± 4.1 | 10.4 ± 6.1 | 21 ± 6 | 12 ± 3.7 |
| SRP_ox_ | mmol kg^-1^ | 2.8 ± 0.6 | 8.3 ± 2.3 | 2.9 ± 1 | 12.7 ± 2.9 | 2.4 ± 0.3 | 7 ± 3.6 | 3.4 ± 0.8 | 9.6 ± 3.9 |
| P_Olsen_ | mmol kg^-1^ | 177 ± 92 | 1861 ± 447 | 75 ± 52 | 1549 ± 230 | 100 ± 2 | 1364 ± 500 | 166 ± 41 | 935 ± 433 |
| SRP_H2O_ | µmol kg^-1^ | 3.5 ± 3 | 8.9 ± 1.3 | 3.9 ± 2.4 | 6.5 ± 2.3 | 2.1 ± 0.3 | 8 ± 2.3 | 4.2 ± 1.6 | 9.7 ± 0.3 |
| CMB | µmol g^-1^ | 88 ± 50 | 12.5 ± 2 | 175 ± 56 | 16 ± 3 | 182 ± 30 | 16.4 ± 6.8 | 295 ± 179 | 12.3 ± 6.5 |
| PMB | µmol g^-1^ | 2.11 ± 0.99 | 0.39 ± 0.24 | 4.22 ± 2.77 | 1 ± 0.43 | 4.58 ± 0.65 | 0.43 ± 0.2 | 10.7 ± 7 | 0.39 ± 0.17 |
| NMB | µmol g^-1^ | 11.9 ± 7.5 | 2 ± 0.4 | 23.3 ± 5.2 | 2.8 ± 1 | 23.9 ± 3.1 | 3.2 ± 1.1 | 34.2± 19.8 | 2.4 ± 1.3 |
| CMB:NMB |  | 7.9 ± 1.3 | 6.3 ± 0.3 | 7.5 ± 1.4 | 5.8 ± 1.0 | 7.6 ± 0.7 | 5 ± 0.4 | 8.5 ± 0.3 | 5.6 ± 0.5 |
| δ 13C | ‰ | -25.91 ± 0.3 | -24.9 ± 0.5 | -26.06 ± 0.4 | -23.8 ± 0.15 | -25.9 ± 0.12 | -23.9 ± 0.24 | -25.93 ± 0.21 | -24.17 ± 0.51 |

**Literature cited:**

Kopáček J., Kaňa J., Šantrůčková H. (2006) Pools and composition of soils in the alpine zone of the Tatra Mountains. Biologia 61: S35–S49.
